# International Collaboration: Promises and Challenges

**DOI:** 10.5041/RMMJ.10196

**Published:** 2015-04-29

**Authors:** R. Jay Widmer, Jocelyn M. Widmer, Amir Lerman

**Affiliations:** 1 Division of Cardiovascular Diseases, Department of Internal Medicine, Mayo Clinic and College of Medicine, Rochester, MN, USA;; 2 Department of Urban and Regional Planning, College of Design, Construction and Planning, University of Florida, Gainesville, FL, USA

**Keywords:** Clinical trial, collaboration, international, train the trainer

## Abstract

We currently face a myriad of grand global challenges in fields such as poverty, the environment, education, science, and medicine. However, our current means of dealing with such challenges has fallen short, and ingenious solutions are required to overcome the inherent resistance to progress toward ameliorating such difficulties. Here, we highlight the promises and challenges of international collaboration in achieving success toward these trials. We note prior successes in fields such as education, medicine, science, and environmental issues made to date, yet at the same time we do note deficiencies and shortcomings in these efforts. Hence, the notion of international collaboration should be strengthened and encouraged by governments, non-profit organizations, and others moving forward using creative means to bring talented teams together to tackle these challenges across the globe.

From flat, to round, back to flat, and now even considered hyper-connected, the world has (r)evolved to become a prolific pitch rife with collaborative ideas and practices opportunistically aimed at improving circumstances for humanity while systematically defying physical borders. Collaboration between entities in different countries has steadily increased over the past two decades secondary to improved mobility of information, ideas, and people across the globe. The promise of international collaboration is evident on a daily basis, with recent successes including the mobilization of resources to areas affected by the catastrophic natural disasters in Haiti (2010 earthquake) and Japan (2012 tsunami), the recent explosion of digital technology in the health care field, and even the re-emergence of Ebola in West Africa and the rapid global response to prevent a worldwide pandemic. Despite the imminent and present potential, the role of international collaboration needs to grow to meet the worldwide demands in business, trade, education, and health in order to address many of the global grand challenges that exist at the intersection of disciplines, geographies, cultures, economies, and policies.

While the developed world tends to lead traditional metrics in terms of numbers of partners and publications stemming from international relationships,^1^ emerging developing states such as Israel and Singapore have opportunistically advanced their economies based on collaboration through technology (among other inputs). Previously, geographic borders restricted scientific collaboration—an island syndrome of sorts. In Singapore, the evolution from manufacturing hard drives in the 1980s into developing data storage technology and programs has increased this city-state’s economy over 250-fold since 1965. Israel has also taken advantage of globalization, being referred to as “Startup Nation” in the 2009 best-selling book of the same name by Dan Senor and Saul Singer.^2^ In addition to cultural and geographic influences, many agree military participation and an opportunistic (albeit stable) government contribute to such a vibrant entrepreneurial economy.^3^ In fact, the US–Israel Binational Industrial Research and Development Fund has not only been successful at fostering business proposals between US and Israeli companies, but is one of the few sustainable funding sources for international collaboration that withstood the recent global recession and continues to thrive with advances in medicine, energy, and technology.

Among national governmental interest in international co-operation is the ability to break barriers between countries that might not have open and collegial relations using affiliations surrounding common scientific interests that are capable of transcending even the most deep-seated political rifts. Membership to communities of science and communities of practice has the potential to be universal. Collaborators can work behind the scenes on projects in an effort to establish small commonalities while promoting focused human advancements. One prominent example is that of Nobel Peace Prize-winning physician, James Muller, MD, who co-founded International Physicians for the Prevention of Nuclear War. This movement began as he, as a member of the faculty of Harvard Medical School, collaborated with Soviet physicians and scientists during the Cold War. These ties can extend to build lasting international relationships, upon which other diplomatic ties can be based. Additionally, governments can maximize their investment in certain projects by assembling multiple and diverse teams with various strengths to facilitate enhanced advancement of knowledge and discoveries along the continuum of “science to practice to policy.”^4^

As diversity in both geography and gender—and the general empowerment of women globally—has become paramount in international collaboration, nowhere is the rise of female enablement more evident than in sub-Saharan Africa. The growing emergence of female leadership both as presidents and First Ladies has come to the forefront in nations such as Liberia where the leadership of Ellen Johnson Sirleaf has successfully navigated the Ebola outbreak. The female leadership has empowered others at the most remote village level to take responsibility for the health and well-being of themselves. A stellar example of this is the dramatic success of The US Presidents Emergency Plan for AIDS Relief (PEPFAR) which has corollary effects on the population scale with fewer children orphaned by HIV/AIDS and susceptible to the social determinants of health that impact orphans especially in sub-Saharan Africa. This has further impact on the next generation through improvements in maternal and child health that not only target survival, but mean that newborns, infants, and young children thrive through the promotion of exclusive breastfeeding, increased mother-and-child interaction during pregnancy and after birth, and improved nutrition through the first 1,000 days of life. These children now have the opportunity to reach their full developmental potential^5^ across measures of health, nutrition, and education.

Perhaps the most established and varied means by which international collaboration is evident is in the field of education. For centuries individuals have been traveling abroad to learn new skills to bring back to their homeland. Certainly world travel and the consequent learning was a substantial factor in the success of Drs Charles and Will Mayo in the early twentieth century, as they assembled a cutting-edge surgical practice ensconced in farmland, in spite of the lack of a thriving metropolis. While unwittingly setting precedent, the Mayo brothers were utilizing a recently trending educational model, “train the trainer,” whereby an individual or group in need of a skill is teamed up with an entity possessing more expert knowledge. The learning occurs through various mechanisms from agricultural and vocational skills in the most resource-challenged contexts to micro-lectures usually delivered through the help of the internet among other technological and communicative advances. Yet what is most critical about this model is that knowledge becomes more democratic and decentralized, furthering a participatory process by which the learning continues as those who have been trained by the “expert” quickly transition into resident experts and subsequently train others and disseminate knowledge (Figure 1).

**Figure 1.**
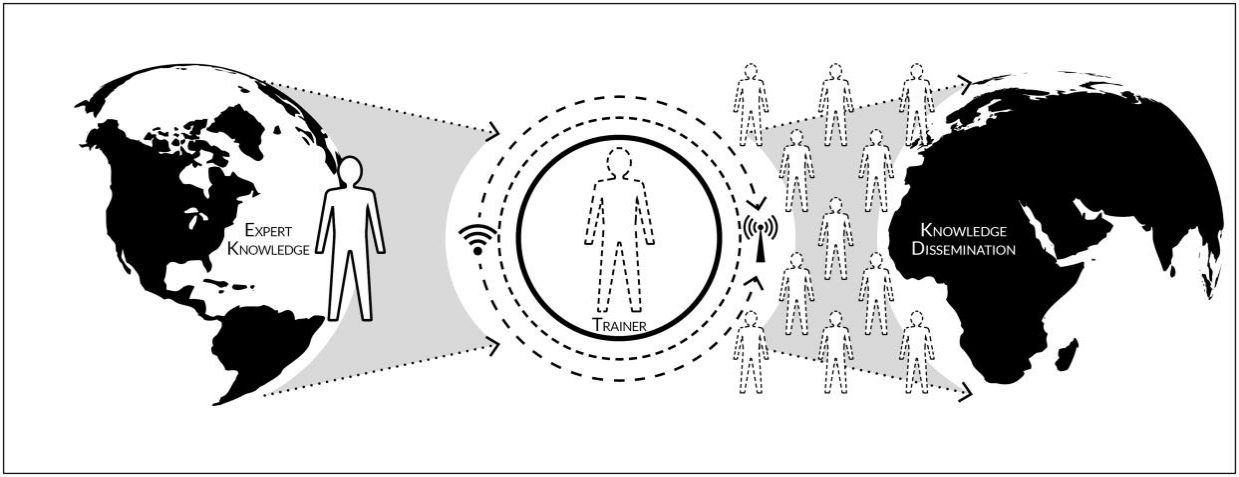
Train the Trainer Conceptual Model.

This allows propagation of knowledge and is increasingly effective in areas of the world lacking in either access to or quality of education (or both) at all levels—but particularly at university and professional levels. With improved mobility and the transfer of people and information, trainees can be educated either at home or abroad, bringing back valuable skills and resources to achieve additional dimensions of mobility: social and economic. This has been successful in many parts of the developing world and has most recently proved effective in engaging an emerging citizenry in Haiti, as it attempts to recover not only from a recent devastating earthquake but also from decades of corruption and political unrest, which have stifled planning and policy measures for the future. Individuals at the most decentralized level of society have been able to become educated in areas such as access, delivery, and protection of water and the corollary public health challenges with only limited direction from various US universities, multi- and bilateral aid organizations, and faith-based groups—and oftentimes facilitated by open-source web sharing platforms such as Google Earth, Google Drive, Dropbox, and Android-based applications used for data collection in the field.^6^

As a growing community of social and public intellectuals emerges in an era of crowd-sourced data collected for studies published in open-access journals, it is important to channel these digital habits that are shaped by and shaping our incessant need to be connected toward international collaborations as the internet allows us to participate and engage in the world around us in productive and meaningful ways. The “train the trainer” model is growing in popularity because it keeps people in the context of the issues they are being capacitated to address, thereby circumventing the potential of brain drain. This model connects the learning with the challenges as they exist rather than removing the learning from the challenges so that the learning becomes only conceptual and theoretical. The “train the trainer” model acknowledges the relationships of interdependence that bind together the units of social organization that make up the global population (nation-states, private organizations, ethnic groups, and civil society movements)^7^ and allows trained professionals to participate and move up in their own economy while alleviating the pressures (health, educational, economic) on the global whole.

Amidst the regional and global risks associated with brain drain, in the past few decades we see an increasing number of trainees coming to the United States for post-graduate education in engineering, business, science, education, and medicine. Estimates from 2012 put the number of foreign students in the US at nearly 800,000, with only 275,000 students from the US traveling abroad for education.^8^ Indeed, this exchange of training, ideas, and education should allow for an explosion of productivity. However, various governmental regulations hinder taking full advantage of these exchanges, particularly government regulations on internet access, online communication mechanisms (e.g. Skype in Pakistan), and social media websites (e.g. China and others^9^). While avenues should be explored to channel resources whereby trainees can practice “at the top of their license”—either at home or abroad—to address those grand challenges that plague a global society, so too should more fundamental challenges associated with government regulations on the internet and the highly regulated emergence of market competition. The trickle-down effect of market competition^10^ has significant implications for global access to training (e.g. Massive Open Online Courses or MOOCs) and collaboration in the form of staying connected when distance and professional obligations can prevent face-to-face contact throughout the life of the collaborative endeavor.

The unbalanced incorporation of global educational resources is clearly evident in the medical field as the Educational Commission for Foreign Medical Graduates reports that over 170,000 foreign medical graduates achieved certification from 1998 to 2008.^11^ In fact, over 9,000 certifications were issued in 2013 from 1,089 medical schools located in 143 countries or territories, with an overwhelming majority being from medical schools in India and Grenada. Interestingly, nearly 30% of foreign medical graduates receiving certification in 2013 were US citizens, highlighting the need for medical education reforms in the US.

Despite attractive educational and occupational opportunities, the US and the rest of the developed world cannot simply stand by and passively recruit international academics. Lucrative offers from both the developed and developing world have increased the level of competition for talented trainees among centers of higher education. Economic impact is a chief driver as this component can drive as much as £10 billion for the UK economy.^12^ Furthermore, employers are increasingly looking for globally minded graduates with cross-cultural competencies or cultural intelligence. As the level of competition increases, an alternative strategy should be to work for a collaborative approach to intercontinental educational endeavors. Instead of competing with the outside world for students, a collaborative approach could foster an environment whereby domestic students study alongside students from other countries to produce substantially more globally trained students across multiple fields and disciplines. While some mechanisms for these collaborations do exist (especially US-based efforts to internationalize university curricula and the recent trend to open international program locations in the Middle East^13^), funding becomes a large barrier, and metrics to determine the efficacy are difficult to establish and measure. Nevertheless, the promise for international collaboration in the educational field will, and should, continue while devising clever means to bypass barriers established by governments and traditional academia—where ultimately governments and traditional bricks-and-mortar university and/or professional programs benefit from these collaborations.

Nowhere is international collaboration more promising or necessary than in the field of medicine, specifically medical research. Large, multicenter, multinational, randomized clinical trials have become an evidence-based standard to validate new treatments or pharmaceuticals and to discern the most efficacious therapies in standard practice. Certainly the EU has been calling for their own institutions to be international leaders in medical science collaboration for over a decade^14^ and has stepped to the forefront in large, international clinical trials. To accomplish such multi-year projects, large international consortia must successfully be assembled. Furthermore, the subgroup analyses of these trials offer important insight into clinical dilemmas and can even alter standards of clinical practice. An initial subgroup analysis by country in the PLATO trial which compared ticagrelor to clopidogrel in acute coronary syndromes^15^ revealed an increased adverse event rate in US participants who were on doses of aspirin over 81 mg daily. This caused the guideline-writing committee to pause and simplify aspirin dosing for secondary prevention to 81 mg daily in opposition to 162 mg or 325 mg daily. Without incorporating a multinational approach, and subsequent subgroup analyses, these insights would never have been reached, and thus we have maximized secondary prevention therapy for patients while minimizing adverse risks.

Another recent example of the benefits of multinational studies and subgroup analyses rests in the TOPCAT trial which studied the effect of aldosterone antagonism in patients suffering from heart failure with preserved ejection fraction.^16^ Despite an overall neutral trial result, subgroup analyses in patients from Eastern Europe introduced heterogeneity to the results in that that those from Russia and Georgia had different outcomes. Furthermore, far fewer patients in Russia and Georgia reached the primary outcomes compared to those from the Americas. These subgroup analysis results have been hypothesis-generating and even spawned new evaluations of the prevalence and characteristics of heart failure around the globe as well as the movement toward standardizing cardiovascular care around the world to erase these disparities.^17^ Global partnerships are also evident in ischemic heart disease, attempting to answer the questions surrounding optimal medical therapy versus an invasive strategy in the ISCHEMIA trial.^18^ This large, National Institutes of Health (NIH)-funded trial will incorporate over 400 international centers targeting 8,000 patients. Studies inclusive of these regional variations will be crucial in elucidating globally effective therapies for cardiovascular disease, and thus require improvements on already successful international collaborations. Funding sources such as the NIH and National Heart Lung and Blood Institute are to be congratulated on such studies, and at the same time challenged to continue fostering these alliances with other governmental agencies and industry sponsors. Care will have to be taken to evaluate these partnerships, not only in terms of study outcomes and metrics, but also in the efficiency and manner in which the overall project was undertaken to provide a springboard for future ventures. The growing worldwide epidemic of cardiovascular disease underscores the importance for international collaborations to address complex, global grand challenges such as the impact that cardiovascular disease is having not only on developed nations but also on low- and middle-income countries where it accounts for nearly 30% of all deaths.^19^ While the rhythms of development are contributing to the increased incidence of cardiovascular disease globally, the solution may lie in engaging the very context giving rise to the problem. As peer-to-peer collaborations are improving agricultural production, yield, market conditions, and nutrition through mobile connectivity and banking at the most basic levels in sub-Saharan Africa,^20^ why not apply the same real-time approach to collecting data toward improving outcomes associated with cardiovascular disease by connecting patients all over the world with highly trained physicians?^21^,^22^

The Mayo Clinic Center for Social Media has taken the lead in driving the global conversation on (and through) social media in health, medicine, and science. Clearly social media help identify potential projects and facilitate the flow of ideas. This was highlighted by a recent report from The European Society of Cardiology Congress in 2014 where it was found that over 50% of conference attendees were active on some social media venue during the conference.^23^ This sentiment was continued with a provocative, albeit neutral, trial showing no apparent benefit in website visits among articles randomized for promotion via social media.^24^ Indeed, the role for social media and increased internet use has facilitated global collaboration, but, again, the metrics used to measure productivity are yet to be properly vetted, let alone be put into use.^25^

Despite the immense progress in international collaboration in just a few short years, challenges remain. Connecting potential collaborators, identifying funding sources, executing and sustaining the project, and evaluating both the project and partnership are all potential barriers to a successful project. Here we point out successes seen in both popular and scientific press over the past few years in an effort to spur on future affiliations. With a recovering, yet tenuous, global economy, and a plethora of global complexities that outpace any empirical advancement in science, there is an essential demand for large, international collaborations to confront the grand challenges we face today. Fortunately, we have been given the instruments and reasonably favorable conditions to accomplish these feats, as the world continues to (r)evolve at an unprecedented rate of change.

## Abbreviations:

NIH: National Institutes of Health.
